# Impact of overactive bladder on retrograde ejaculation

**DOI:** 10.1590/S1677-5538.IBJU.2018.0225

**Published:** 2018

**Authors:** Ahmet Salvarci, Mehmet Karabakan, Aliseydi Bozkurt, Erkan Hirik, Binhan Kagan Aktaş

**Affiliations:** 1 Department of Urology, Novafertile IVF Centers and Konya Hospital, Konya, Turkey;; 2 Department of Urology, Mersin Toros State Hospital, Mersin, Turkey;; 3 Department of Urology, Erzincan University, Mengücek Gazi Research and Training Hospital, Erzincan, Turkey;; 4 Department of Urology, Ankara Numune Research and Training Hospital, Ankara, Turkey

**Keywords:** Urinary Bladder, Overactive, Ejaculation, Urodynamics

## Abstract

**Purpose:**

To evaluate the impact of overactive bladder disorder on patients diagnosed with retrograde ejaculation.

**Materials and Methods:**

Retrospective analysis of prospective collected database made. Questionnaires conducted in urology polyclinics in five different centers. Main Outcome Measure(s): International Index of Erectile Function - 5 (IIEF - 5), Overactive Bladder 8 - Question Awareness Tool (OAB - V8), urodynamics, semen analysis. The participants of the study were n = 120 patients. There was retrograde ejaculation (RE) in only n = 47 patients (non / minimal symptomatic patients), n = 73 patients had RE and overactive (OAB) complaints (symptomatic patients) and received anticholinergic treatment (trospium), n = 37 control group patients who only had OAB and received an anticholinergic.

**Results:**

While no difference was observed in overactive bladder examination and urodynamic values between the non / minimal symptomatic group and the symptomatic group (p > 0.05), sperm was detected and identified as fructose positive in post - ejaculation urine in the symptomatic group. Thus, it was possible to demonstrate the differences between symptomatic patients and non - symptomatic patients. Consequently, following three - month daily treatment with trospium 30 mg 2 x 1 in the control group and the symptomatic group, it was observed that an evident increase was observed in the sperm count and ejaculate volume in the symptomatic group and that no change was observed in the control group (p < 0.05).

**Conclusion:**

This clinical study is the first of its kind in terms of revealing the coexistence of RE with OAB upon performing urodynamics and showing that treatment is possible in selected patients.

## INTRODUCTION

Overactive bladder (OAB) is a common condition affecting quality of life. OAB is often defined by lower urinary tract symptoms (LUTS), such as frequency, nocturia and urgency with or without incontinence (1). Irwin et al. reported that in 2006 the general prevalence of OAB in Euro-pe and Canada was 10.8%, but the frequency of LUTS symptoms in men was much higher (62.5%) (2). Stewart et al. found the OAB prevalence in men to be 16.0% (3). In another study based on the results of a large epidemiological population - representative survey conducted in the United States, United Kingdom and Sweden to investigate the epidemiology of LUTS (EpiLUTS), the prevalence of urinary frequency and urgency in the female and male adults aged over 40 in the United States was reported to range from 15.8% to 27.2% (4). Furthermore, it was reported in various studies that LUTS are associated with erectile dysfunction (ED) (5, 6) as well as reduced sexuality (7, 8), premature ejaculation (PE) (8) and partial or complete ejaculatory loss (6, 8). In another paper reporting the results from EpiLUTS, in addition to ED and PE, ejaculatory difficulty or loss was detected in 22.3% of the patients diagnosed with OAB (9).

Ejaculation is a complex process controlled by the interaction between peripheral, cerebral, spinal sensory and motor neural pathways (10). Neurons in the cortex, thalamus, hypothalamus, midbrain and pons play a role in the ejaculation process, but their functions are not yet clear (11, 12). During ejaculation, the bladder neck closes and the external urethral sphincter opens. This mechanism, coordinated by sympathetic fibers, prevents retrograde ejaculation into the bladder (13, 14). In retrograde ejaculation, all components of ejaculatory reflex continue to function normally with the exception of the closure of the bladder neck. Due to anatomic or physiologic factors, the bladder neck may not close, resulting in retrograde ejaculation. This may be caused by congenital abnormality, spinal trauma, retroperitoneal lymph node dissection, diabetes and bladder neck surgery, many medications or idiopathic conditions (15, 16). When the contraction of the internal sphincter is not sufficient, the resulting low pressure causes semen to flow backwards into the bladder.

In this study, patients presenting with the complaint of retrograde ejaculation were evaluated in terms of the presence of OAB and the relationship between these two conditions was investigated.

## MATERIAL AND METHODS

The participants of the study were 120 patients who presented with retrograde ejaculation (RE) between January 2013 and October 2017 in five different centers. There was retrograde ejaculation without overactive bladder symptoms (RE) in only 47 patients who received no therapy (non / minimal symptomatic patients) while 73 patients had RE and overactive (OAB) complaints (symptomatic patients) and received anticholinergic treatment (trospium). The patients in the control group were 37 patients who only had OAB and no RE. The control group patients were selected randomly among patients who were admitted to the polyclinic due to OAB complaint and used an anticholinergic for at least 3 months or a longer period.

Our primary objective was to evaluate the urodynamics and ejaculation outcomes of patients who had both RE and OAB symptoms (symptomatic patients) and patients who only had RE (non / minimal symptomatic patients) and thus to show the differences between symptomatic and non -symptomatic patients.

Our secondary objective was to compare the semen analyses and urodynamic values of symptomatic patients treated with an anticholinergic and the control group patients who only had OAB, had no RE and received an anticholinergic, to evaluate the changes in semen volumes and numbers before / after treatment between the symptomatic group and the control group and ultimately to check whether there were any significant differences in the symptomatic group patients compared to the control group in order to verify whether the treatment was significant (Tables 1 and 2). The ethical approval for the study was obtained from the local ethics committee of the institution (Decision No. 1 / 02 adopted in Session 1 on 08 / 01/2015 by the Ethics Committee of the School of Medicine of Erzincan University). Furthermore, all authors of the paper received a permit from their relevant healthcare institution.

**Table 1:** Semen parameters and urodynamic results before and after anticholinergic treatment in patients proven to have coexisting RE and OAB (n=73 symptomatic patients).

	Pre-treatment		Post-treatment		
	Mean ± s.d.	Median	Mean±s.d.	Median	p
OAB-V8 Score	24 ± 2.7	24	6.6±1.6	6.0	0.000W
First urge to void	78.6 ± 9.9	78	130.1±16.3	133	0.000W
Qmax(mL/s)	22.1 ± 4.6	21	21.7±2.2	21.0	0.912W
Maximal bladder pressure (cm H_2_0)	75.4 ± 12.2	76	47.2±8.2	48.0	0.000W
Bladder capacity (mL)	330.0 ± 19.9	326	378.6±21.1	379	0.000W
Number of sperm (x10^3^)	0 ± 0	0	11400±5480	10400	0.000W
Ejeculat Volume	0.4 ± 0.4	0.3	2.6±1.3	2.3	0.000W

**w Wilcoxon test**

**Normal first urge to void value:** 150-200/mL; **Normal bladder capacity value:** 300-600 /mL; **Normal ejaculate volume (WHO 2010):** >1.5 mL; **Number of sperms (x106)/mL (WHO 2010):** >15x106/mL; Overactive Bladder 8-Question Awareness Tool

**Table 2:** Patients with retrograde ejaculation and overactive bladder symptoms and receiving anticholinergic treatment (n = 73 symptomatic patients) and patients with only overactive bladder.

		Symptomatıc Patientsw		Control Grup		
		Mean±s.d.	Median	Mean±s.d.	Median	p
Age		36.7±7.7	36.0	34.9±6.1	35.0	0.325^m^
Fasting blood sugar		95.1±8.2	96.0	93.8±7.3	95.0	0.627^m^
**OAB-v8 Score**						
	Pre-treatment	24.01±2.7	24.0	24.8±2.8	25.0	0.154^m^
	Post-treatment	6.6±1.6	6.0	6.5±1.6	6.0	0.801^m^
**Qmax(mL/sn)**						
	Pre-treatment	22.1±4.6	21.0	22.8±4.9	22.0	0.566^m^
	Post-treatment	21.7±2.2	21.0	21.3±1.9	21.0	0.495^m^
**first Urge to void**						
	Pre-treatment	78.5±9.9	78.0	80.7±9.0	76.0	0.310^m^
	Post-treatment	130.1±16.3	133.0	130.1±21.2	135.0	0.502^m^
**Bladder capacity(mL)**						
	Pre-treatment	330.0±19.9	326.0	323.5±17.3	319.0	0.118^m^
	Post-treatment	378.6±21.1	379.0	375.2±21.6	370.0	0.516^m^
**Max.bladder pressure(cm H_2_O)**						
	Pre-treatment	75.4±12.2	76.0	76.2±10.3	77.0	0.776^m^
	Post-treatment	47.7±8.2	48.0	47.5±7.0	48.0	0.902^m^

m **Mann-Whitney U test**

**Normal first urge to void value:** 150-200/mL; **Normal bladder capacity value:** 300-600 /mL; **Normal ejaculate volume (WHO 2010):** >1.5 mL; **Number of sperms (x106)/mL (WHO 2010):** >15x106/mL; **Overactive Bladder 8-Question Awareness Tool (OAB-V8), Maximum urine flow rate (Qmax) (Campbell Urology, 2016).**

RE diagnosis was made based on the presence of fructose and spermatozoa in the centrifuged post - ejaculation urine samples. Semen analyses were conducted following a sexual abstinence of a minimum of 3 days. Fructose and sperm levels were checked again in the post - ejaculation urinalysis after three months and the efficiency of the treatment was examined. Is post - ejaculation semen in the urethral residue? Or the semen entering the bladder? In order to fully distinguish this, we have included into the study patients who had no sperm detected in their semen analysis. Yet, there have been patients identified with pellette sperms between the microscope slides or after centrifuging (cryptozoospermia). We had no cutoff evaluation for this. Furthermore, we made sure that there was no sperm in the ejaculate in order to discuss the neurological mechanisms and the efficacy of treatment and to present RE which is really associated with OAB.

Patients with a psychiatric, psychological or neurologic disorder or history of alcoholism or other substance abuse, which was clearly the cause of anejaculation, as well as patients that were taking any medication which could affect their sexual functions or who were on drugs (alpha-blockers, antidopaminergics, yohimbine, ergot alkaloids, finasteride, etc.) were excluded from the study. The medical and sexual history of the patients was obtained.

In addition, all patients were evaluated in terms of the results of their physical examination, urinary, transrectal ultrasonography (TRUS), tests on total blood count and biochemical parameters, uroflowmetry (UFM) and Overactive Bladder 8 -Question Awareness Tool (OAB - V8).

The patients were asked to complete the International Index of Erectile Function - 5 (IIEF - 5) form to assess their current and past sexual dysfunction. IIEF - 5 scoring: The IIEF - 5 score is the sum of the ordinal responses to 5 items. 22 - 25: No erectile dysfunction, 17 - 21: Mild erectile dysfunction, 12 - 16: Mild to moderate erectile dysfunction, 8 - 11: Moderate erectile dysfunction, 5-7: Severe erectile dysfunctqion.

A further pressure flow study was performed on patients who had OAB symptoms. OAB - V8 is a self - administered questionnaire containing eight questions designed to determine how bothered the patients are with their OAB symptoms, such as frequency, nocturia, urgency and urge incontinence (17). Patients responses were graded on a 6 - point Likert scale ranging from 0 (not at all) to 5 (a very great deal), with the maximum possible score being 40. The bladder values were checked via urodynamic testing in patients considered to have OAB in the OAB - V8 questionnaire. Thus, OAB diagnosis was confirmed with urodynamics and the flow of the treatment could be monitored.

All patients having OAB + RE symptoms were started on trospium chloride in doses of 30 mg twice daily and asked to return for a follow-up after three months. In the follow-up, the patient's ejaculatory functions and LUTS symptoms were evaluated again through sperm analysis, OAB - V8 questionnaire, urodynamics and UFM (Tables 1–3).

**Table 3:** Semen parameters and urodynamic results before and after anticholinergic treatment in patients proven to have coexisting RE and OAB (n = 73 symptomatic patients).

		Symptom Patients		Control Group		
		Mean±s.d.	Median	Mean±s.d	Median	P
**Ejeculate Volume**						
	Pre-treatment	0.4±0.4	0.3	3.1±1.0	3.0	**0.000^mm^**
	Post-treatment	2.6±1.3	2.3	3.2±1.0	3.0	**0.001^mm^**
	Pre-Post Difference	2.3±1.4	2.2	0.1±1.7	0.2	**0.000^mm^**
	Pre-Post Difference p value	0.000^ww^		0.694ww		
**IIEF-5**						
	Pre-treatment	19.1±1.8	19.0	24.3±1.6	24.0	**0.000^mm^**
	Post-treatment	16.5±1.7	16.0	24.3±1.5	24.0	**0.000^mm^**
	Pre-Post Difference	-2.6±2.5	-3.0	0.±0.2	0.0	**0.000^mm^**
	Pre-Post Difference p value	**0.000ww**		0.924ww		
**Sperm Count (x10^6^)**						
	Pre-treatment	0.00±0.0	0.0	19.1±5.2	19.2	**0.000^mm^**
	Post-treatment	11.1±5.5	10.4	18.9±4.8	18.9	**0.000^mm^**
	Pre-Post Difference	11.1±5.5	10.4	-0.1±0.7	0.0	**0.000^mm^**
	Pre-Post Difference p value	**0.000ww**		0.456ww		

**^m^ Mann-Whitney U/ ^w^ Wicoxon test**

**Normal first urge to void value:** 150 - 200 / mL; **Normal bladder capacity value:** 300 - 600 / mL; **Normal ejaculate volume (WHO 2010):** > 1.5 mL. **Number of sperms (x10^6^) / mL (WHO 2010):** > 15 x 10^6^ / mL; Overactive Bladder 8 - Question Awareness Tool (OAB - V8), Maximum urine flow rate (Qmax) (Campbell Urology, 2016)

### Statistical analysis

Mean and standard deviations, median, minimum and maximum value frequencies and percentages were used for descriptive statistics. The distribution of variables was checked with Kolmogorov - Smirnov test. Mann - Whitney U test was used for the comparison of quantitative data. Wilcoxon test was used for the repeated measurement analysis. SPSS 22.0 was used for statistical analyses.

## RESULTS

The mean age was calculated as 36 years in symptomatic patients (n = 73; with RE + OAB and receiving treatment), 42 years in non/minimal symptomatic OAB patients (n = 47; with only RE and receiving no treatment; this is only the cohort group and not the placebo group) and 36 years in control patients (n = 37; with only OAB symptoms, no RE and receiving treatment).

All the patients experienced RE complaints for more than one year (2 to 6 years). The primary complaints were reduction in ejaculate volume and frequency, nocturia. Furthermore, there were also some who recorded decrease in libido and frequency of intercourse. There were some who reported anejaculation. There were others who recorded that they had frequency and urinated with a sense of urgency and had no semen occasionally and experienced performance anxiety due to dry intercourse.

The results of the routine biochemical and urinary microbiology tests were normal in all patients. There was no pathology detected in the TRUS and ultrasonography of the urinary system in all patients. The blood glucose, ejaculate volume, Q - max did not display a significant difference in the symptomatic, non - symptomatic and control groups (p > 0.05). The IIEF - 5 score was lower in the non - symptomatic group. When this was questioned, it was reported that this was the reason for retrograde ejaculation in 9 patients while the other patients recorded that they experienced work and environmental stress or that the spouse had psychological and additional familial problems. There were some who said that this was due to loss in hardness during intercourse or premature ejaculation. No treatment was applied to the group which only had RE. The OAB V8 score of the patients with symptomatic patients compared to those without no/minimal OAB symptom was significantly higher (p < 0.05).

### Semen parameters and urodynamic results before and after anticholinergic treatment in symptomatic patients (n = 73; with RE + OAB and receiving treatment).

Symptomatic (n = 73) patients were started on trospium chloride in doses of 30 mg twice daily for three months. In these n = 73 patients, a statistically significant difference was observed between the pre-treatment and post - treatment values of the first urge to urinate, ejaculate volume and maximum bladder capacity. The OAB - V8 score was significantly reduced compared to the score prior to treatment in the symptomatic group patients (p < 0.05). The initial mean volume of ejaculate was 0.7 ± 1.2 mL. At three months after treatment, the ejaculate volume of n = 73 symptomatic patients was calculated as 2.6 ± 1.3 mL and sperm was present in the ejaculate. This indicated a significant increase in the ejaculate volume of all n = 73 patients (p < 0.05) (Table-3). While the mean sperm count in the pre - treatment ejaculate was 0.0 ± 0.0 x10^6^ / mL in n = 73 OAB patients, their mean post - treatment sperm count was measured as 11.4 ± 5.48 x10^6^ / mL (p < 0.05) (Table-3). Cystometric examination showed that symptomatic patients had a bladder volume of 330.0 ± 19.9 mL and the first urge to urinate at a bladder filling of 78.5 ± 9.9 mL. In symptomatic patients, the first urge to void value was also reduced significantly after treatment compared to the pre - treatment value (p < 0.05). The post - treatment Q - max value displayed no significant change compared to the pre - treatment value (p > 0.05). The maximum post - treatment bladder pressure was significantly decreased compared to the level prior to treatment (p < 0.05). The bladder capacity after treatment increased significantly compared to the period prior to treatment (p < 0.05). The post - treatment sperm count in the ejaculate increased significantly compared to the level prior to treatment (p < 0.05). The ejaculate volume value after treatment increased significantly compared to the pre - treatment value (p < 0.05) (Table-3). No sperm was detected in the urine following semen analyses in the symptomatic group following treatment and fructose was detected as negative.

Dryness of the mouth was identified in eight patients in relation with the medicine during treatment, while constipation was detected in six patients and nausea was observed in two patients. Nonspecific stomach ache occurred in one patient. The complaints were reduced with symptomatic treatment and no side effect strong enough to lead to discontinuation of treatment was observed.

### Comparison of patients with symptomatic (n = 73; with RE + OAB and receiving treatment) and control patients (n = 37; with only OAB symptoms, no RE and receiving treatment)

No significant difference was obtained in the age of symptomatic patients and the control group and the fasting blood glucose level (p > 0.05). No significant differences occurred in the OAB - V8 score, Q - Max, first urge to void, bladder capacity, maximal bladder pressure before and after treatment in the symptomatic patients and the control group (p > 0.05) (Table-1). No significant change was displayed in the ejaculate volume after treatment in the control group compared to the level before treatment (p > 0.05). The increase in the ejaculate volume in symptomatic patients after treatment was higher than that of the control group (p < 0.05). IIEF - 5 in symptomatic patients before and after treatment was lower than that in the control group (p < 0.05). No significant change occurred in IIEF - 5 after treatment compared to the level prior to treatment in the control group (p > 0.05). The increase in IIEF - 5 after treatment in symptomatic patients was significantly higher than the level in the control group (p < 0.05). The number of sperms before and after treatment in symptomatic patients was significantly lower than that in the control group (p < 0.05). No significant change occurred in the number of sperms after treatment in the control group compared to the number before treatment (p > 0.05). The increase in the number of sperms in symptomatic patients prior to treatment was significantly higher than that in the control group (p < 0.05) (Table-2).

In conclusion, a significant difference was observed before and after treatment between the symptomatic and control groups in terms of the increase in the ejaculate volume, number of sperms and IIEF - 5 (p < 0.05) (Table-2).

## DISCUSSION

This clinical study is the first of its kind in terms of revealing the coexistence of RE with OAB upon performing urodynamics and showing that treatment is possible in selected patients. No clinical observation was identified regarding this topic except where OAB could lead to loss of ejaculation.

### Overactive bladder & Emission

These are mainly composed of the adrenergic, cholinergic, non - adrenergic and non -cholinergic receptors, interstitial cells and the nerves enabling the afferent activity of the bladder (18). The main parasympathetic system responsible for bladder emptying is the main acetylcholine neurotransmitter. The receptors leading to relaxation of the bladder are released with acetylcholine upon neural stimulation of adenosine triphosphate (ATP), inducing contraction via purinergic receptors (19). The receptors leading to relaxation in the bladder are M2 and M4 with muscarinic and presynaptic localization, M2 and M3 receptors in the urothelium, alpha - 2 receptors in the cholinergic nerves, beta - 2 and beta - 3 receptors in the adrenergic detrusor and nitric oxide (NO) in the other sensorial afferent nerves. The receptors leading to contraction in the bladder are M1 and M3 receptors localized in the muscarinic presynaptic localization, P2X1 receptors in the purinergic detrusor, P2X3 receptors in the sensorial afferent nerves and the receptors in the other detrusor, namely histamine, serotonin, leukotriene, neurokinin and angiotensin (19).

The emission in the ejaculation is composed of expulsion and orgasm. The emission is controlled by the sympathetic T10 - L2. The sperm - carrying fluid emanating from vas deferens is accumulated in the posterior urethra, the bladder neck is closed in order to prevent retrograde ejaculation, the external sphincter is relaxed, the pelvic floor muscles contract and relax and the ejaculation takes place under the control of S2 - 4. The emission in the ejaculation is composed of the expulsion, the complex medial preoptic field in the brain controlling the orgasm, paragigantocellular reticular nucleus, cerebellum, stria terminalis, amygdala and the thalamus. Serotonin, endothelin, oxytocin, GABA are nitric oxide stimulant and inhibiting regulators in the ejaculation complex. The spinal ejaculation generator also controls the sympathetic, parasympathetic and motor flow for controlling the emission and expulsion in the spinal cord. The spinal ejaculation generator is under the influence of the medial preoptic field and paragigantocellular reticular nucleus. Emission is controlled by the sympathetic system while expulsion is mostly controlled by the parasympathetic system (11, 18).

### Potential mechanisms in the presence of OAB and RE

The claim that there is no increase in α-adrenoreceptor functions in OAB as accepted by some authors (19, 20) made us think that especially the closure in the bladder neck may be prevented. It was interpreted that especially due to the extreme increase in cholinergic activation and the failure of beta - 2 and beta - 2 receptors in the adrenergic detrusor to compensate for this (insufficient compliance) and thus the occurrence of parasympathetic and sympathetic non - conformity was a provoking power in the prevention of the closure of the bladder neck which contributed to the disruption of emission and expulsion. It is believed that sympathetic and parasympathetic systems are effective via the detrusor and urothelium in all these complicated interactions (11, 18).

### Antimuscarinic choice and how we may have corrected RE in Patients

**Figure 1:**
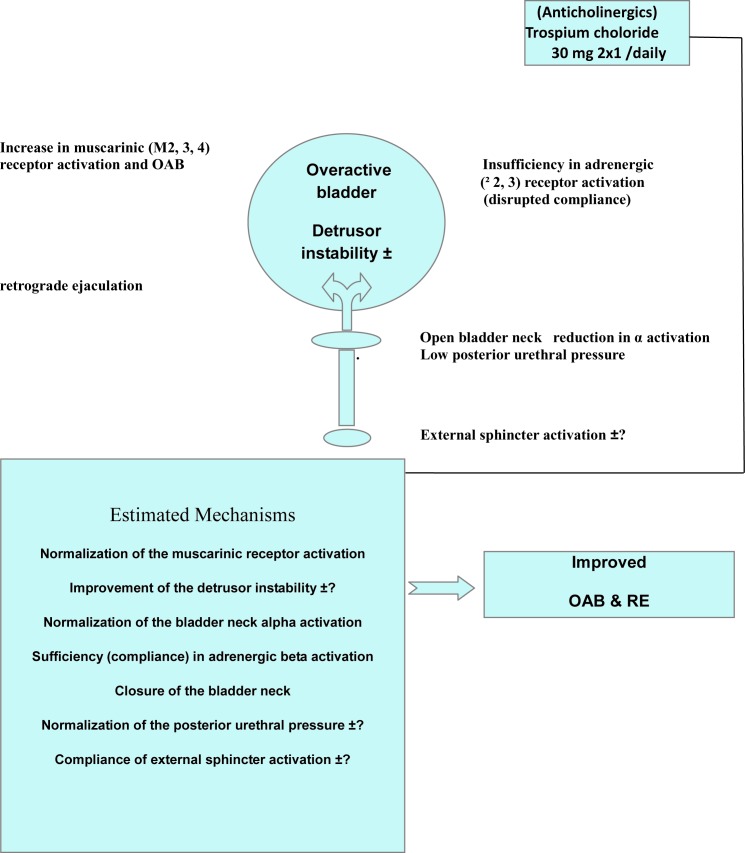
Potential mechanisms and post-treatment potential effects in the co-existence only of RE and OAB.

Still, the clinical effects obtained with antimuscarinic drugs probably comprise a more complicated structure. Due to its low receptor selectivity and high receptor affinity, precedence has been given to trospium in our study. It avails of a non - selective hydrophilic quaternary ammonium structure. It dissolves in water and hardly crosses the blood - brain barrier. It provided an advantage in our study for proving RE concomitantly with OAB and demonstrating its efficacy in treatment (Figure-1). Improvement was achieved in OAB by stopping elevated spontaneous activity in the bladder and disrupted synchronization as well as the intrinsic biochemical variations, correcting the increased sensitivity in membrane instability and achieving a more selective concentration in the bladder. Thus, concentration was achieved only on the bladder neck and the sympathetic and parasympathetic balance in the bladder, reducing urgency attacks, enhancing the bladder capacity, achieving an increase in the voiding volume and improvement in compliance. We believe that due to the observation of at least a low central transition of trospium with the improvement in RE in the symptomatic group and objective improvements in all groups in urodynamic parameters, trospium provides a major contribution in this direction.

We are aware that there are some limitations and deficiencies in the study. Different groups could have been formed with different antimuscarinics in terms of drug use. The impact of overall impact of antimuscarinic drugs could have been observed and compared better. This was considered initially, but focus was placed mostly on the bladder neck. We still believe that the results with different antimuscarinics should be observed. Because, we believe that the co - existence of OAB and RE may have central connections as well as peripheral connections. Another deficiency in our study was the absence of urethral pressure profile (UPP). Due to the fact that the study groups were located in different cities and that not every investigator had adequate experience and equipment on UPP, it was not possible to perform UPP. Obviously, it would have been better to demonstrate the changes in two UPPs. The improvements achieved in RE in the patients via treatment showed us that UPP is not so necessary in practice. Certainly, we don't think that the results obtained are limited with our estimated mechanisms. Because, OAB plays a role in the etiology of RE and it is necessary to investigate the etiology of RE with neurological and multichannel urodynamic exams. Due to the fact that efficiency was achieved in a specific group with OAB + RE with the anticholinergic treatment, our study directed us towards both the bladder and the bladder neck and made us consider more the underlying complex neurological and biochemical mechanisms which supported this treatment. It is clear that there is need for more objective neurophysiological and neurobiochemical studies to unveil neural interactions comprising the micturition center, medulla spinalis, peripheral nerves and the bladder.

The importance of OAB was demonstrated in RE and treated with anticholinergic. It appears to be possible to treat these patients upon differentiating them from others via an adequately performed anamnesis.

### Ethical approval

All procedures performed in studies involving human participants were in accordance with the ethical standards of the institutional and / or national research committee and with the 1964 Helsinki declaration and its later amendments or comparable ethical standards.

Informed consent: Informed consent was obtained from all individual participants included in the study.
